# The anticipating value of PLK1 for diagnosis, progress and prognosis and its prospective mechanism in gastric cancer: a comprehensive investigation based on high-throughput data and immunohistochemical validation

**DOI:** 10.18632/oncotarget.21438

**Published:** 2017-09-30

**Authors:** Peng Lin, Dan-Dan Xiong, Yi-Wu Dang, Hong Yang, Yun He, Dong-Yue Wen, Xin-Gan Qin, Gang Chen

**Affiliations:** ^1^ Department of Medical Ultrasonics, First Affiliated Hospital of Guangxi Medical University, Nanning, Guangxi Zhuang Autonomous Region 530021, P. R. China; ^2^ Department of Pathology, First Affiliated Hospital of Guangxi Medical University, Nanning, Guangxi Zhuang Autonomous Region 530021, P. R. China; ^3^ Department of Gastrointestinal Surgery, First Affiliated Hospital of Guangxi Medical University, Nanning, Guangxi Zhuang Autonomous Region 530021, P. R. China

**Keywords:** PLK1, gastric cancer, prognosis, bioinformatics analysis, immunohistochemistry

## Abstract

Polo-like kinase 1 (PLK1) is a multi-functional protein and its aberrant expression is a driver of cancerous transformation and progression. To increase our understanding of the clinical value and potential molecular mechanism of PLK1 in gastric cancer (GC), we performed this comprehensive investigation. A total of 25 datasets and 12 publications were finally incorporated. Additional immunohistochemistry was conducted to validate the expression pattern of PLK1 in GC. The pooled standard mean deviation (SMD) indicated that PLK1 mRNA was up-regulated in GC (SMD=1.21, 95% CI: 0.65-1.77, P< 0.001). Similarly, the pooled odds ratio (OR) revealed that PLK1 protein was overexpressed in GC compared with normal gastric tissue (OR=12.12, 95% CI: 5.41-27.16, P<0.001). The area under the curve (AUC) of the summary receiver operating characteristic (SROC) curve was 0.86. Furthermore, our results demonstrated that GC patients with PLK1 overexpression were significantly associated with unfavorable overall survival (HR =1.54, 95% CI: 1.30–1.83, P<0.001), lymph node metastasis (OR = 1.78, 95% CI: 1.13–2.80, P=0.013) and advanced TNM stage (OR=1.48, 95% CI: 1.02-2.15, P=0.038). Altogether, 100 similar genes were identified by Gene Expression Profiling Interactive Analysis (GEPIA) and further with gene-set enrichment analysis. These genes were related to gene ontology (GO) terms and Kyoto Encyclopedia of Genes and Genomes (KEGG) pathways relevant to the cell cycle. Gene set enrichment analysis (GSEA) indicated that PLK1 is associated with various cancer-related pathways. Collectively, this study suggests that PLK1 overexpression could play vital roles in the carcinogenesis and deterioration of GC via regulating tumor-related pathways.

## INTRODUCTION

Gastric cancer (GC) is the fourth leading human cancer, and it ranks as the second most common cause of tumor-related mortality all over the world, seriously threatening human health [1]. It is estimated that approximately 28,000 newly diagnosed cases and 10,960 deaths will occur in the United States in 2017 [2]. Although diagnostic and therapeutic techniques of GC have made advances over the past decades, the mortality rate is still fairly high due to its aggressive behavior [3–6]. Thus, a number of researchers have focused on probing several molecular biomarkers to modify clinical management strategies and better understand the molecular mechanism of GC [7–9]. Because clinically applicable biomarkers are fairly meager, exploring novel, effective molecular biomarkers to elucidate effective therapeutic targets for GC patients is still imperative.

Polo-like kinase 1 (PLK1), also known as serine/threonine-protein kinase 13(STPK13), is a multi-faceted regulator of the cell cycle [10, 11]. Due to PLK1's broad biological functionality, it is widely deemed as an oncogene and implicated in a broad range of malignant human tumors, including breast [12], liver [13], and colorectal cancers [14]. Prior to our study, increasing evidence has also suggested that dysregulated expressions of PLK1 exerted indispensable functions in GC progression. For example, Otsu H suggested that GC patients with high expression of PLK1 and DNA aneuploidy had inferior survival outcome [15]. Elevated PLK1 promotes GC cell metastasis rates and epithelial-mesenchymal transition by regulating the activation of the protein kinase B pathway [16]. Despite several independent studies providing various valuable perspectives of PLK1 in GC, low numbers of studies have led to a limited ability to uncover the complexity of GC and no meta-analysis to clarify the reliability and extent of its clinical value in GC has been performed.

Here, we collected all available datasets presenting the PLK1 expression pattern and the clinicopathological significance in GC from the Gene Expression Omnibus (GEO), Oncomine, The Cancer Genome Atlas (TCGA) and the literatures. Simultaneously, immunohistochemistry (IHC) staining was performed using clinical specimens from in-house GC patients to validate the expression pattern of PLK1. Subsequently, all-inclusive information related to PLK1 was achieved, and we proceeded with a systematic investigation using a meta-analysis to draw a comprehensive conclusion. Going further still, potential regulation mechanisms of PLK1 were analyzed using bioinformatics methods. Through these means, we unveil a key role for PLK1 as a GC promoter.

## RESULTS

**Figure 1:**
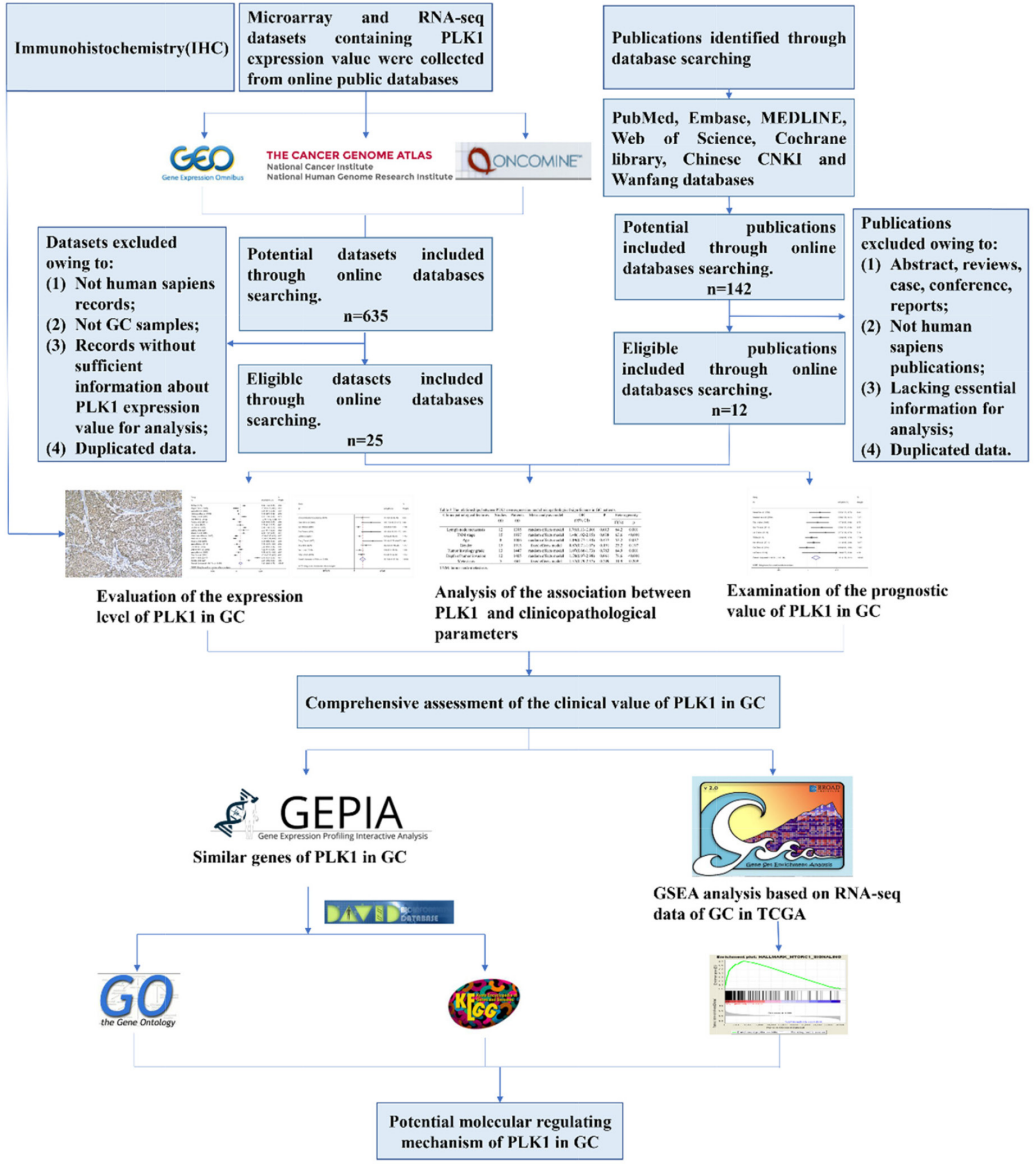
Flow chart of the study design in this investigation

The present study contained several procedures sequentially (Figure 1). After screening and inspection, a total of 25 datasets and 12 publications [15, 17–27] were incorporated into the present study. Among them, 19 datasets and 2 articles offered the expression value of PLK1 mRNA in GC and control groups were included (Table 1). Additionally, a total of 10 datasets and 11 publications that were used for investigating the prognostic value and clinicopathological significance of PLK1 were included and summarized in Table 2.

**Table 1:** Characteristics of microarray and RNA-seq datasets included in the study

First author (publication year)	Country	Data source	Test method/Platform	Cancer group	Normal controls	Mean1±SD1	Mean0±SD0	AUC
TCGA (2017)	USA	TCGA	NR	415	35	11.77±1.13	9.11±1.71	0.915
Hippo Y et al. (2005)	Japan	GEO: GSE2685	Affymetrix GPL80	22	8	7.32±0.43	7.40±0.46	0.426
Wang G et al. (2013)	USA	GEO: GSE29272	Affymetrix GPL96	134	134	6.74±0.53	6.54±0.44	0.594
Holbrook JD et al. (2012)	Singapore	GEO: GSE29998	Illumina GPL6947	50	49	6.18±0.66	5.76±0.72	0.648
Cheng L et al. (2012)	China	GEO: GSE33335	Affymetrix GPL5175	25	25	5.37±0.70	4.05±0.48	0.918
Wu YH et al. (2012)	Singapore	GEO: GSE37023	Affymetrix GPL96	112	39	5.83±0.55	6.24±0.59	0.286
Kang M et al. (2014)	South Korea	GEO: GSE51575	Agilent GPL13607	26	26	7.88±0.98	7.08±0.69	0.743
Lin J et al. (2013)	USA	GEO: GSE52138	Affymetrix GPL96	13	7	6.70±0.60	6.13±0.301	0.769
Liu B et al. (2017)	China	GEO: GSE54129	Affymetrix GPL570	111	21	6.21±0.69	4.63±0.28	0.988
Wang J et al. (2014)	China	GEO: GSE56807	Affymetrix GPL5175	5	5	7.11±0.92	5.11±1.01	0.960
Zhang X et al. (2014)	China	GEO: GSE63089	Affymetrix GPL5175	45	45	6.81±0.90	5.21±0.60	0.934
Yoshizawa JM et al. (2015)	USA	GEO: GSE64951	Affymetrix GPL570	63	31	7.93±1.71	8.00±1.88	0.485
Hao L et al. (2015)	China	GEO: GSE65801	Agilent GPL14550	32	32	6.18±1.69	7.01±2.02	0.378
Shao Q et al. (2016)	China	GEO: GSE79973	Affymetrix GPL570	10	10	6.11±1.12	5.11±0.89	0.800
Wang B et al. (2016)	China	GEO: GSE84787	Agilent GPL17077	10	10	6.40±3.54	5.91±2.99	0.570
Cui J et al. (2013)	USA	Oncomine	Affymetrix GPL5175	80	80	5.73±1.54	4.45±1.55	0.732
D'Errico M et al. (2010)	Italy	Oncomine	Affymetrix GPL570	38	31	9.18±0.71	8.26±0.69	0.818
Wang Q et al. (2010)	China	Oncomine	Affymetrix GPL570	12	15	8.07±1.09	7.39±1.23	0.656
Cho JY et al. (2011)	USA	Oncomine	Illumina GPL6884	71	19	7.24±0.71	7.46±0.80	0.353
Sun YH et al (2014)	China	CNKI	RT-PCR	60	60	0.38±0.07	0.05±0.03	NR
Tang M et al. (2014)	China	CNKI	RT-PCR	60	60	0.19±0.05	0.06±0.01	NR

AUC: area under curve

Note: Mean1±SD1: gastric cancer tissues; Mean0±SD0: non-tumor tissues.

**Table 2:** General characteristics of outcome-related published studies and microarray datasets

Author	Data source	Year	Country	Patients (n)	Test method/Platform	Cut off values	HR estimation	survival analysis	HR (95% CI)
Kanaji S et al.	PMID:16645325	2006	Japan	160	IHC	NR	Multivariate analysis	OS	2.02 (1.10–3.72)
Weichert W et al.	PMID: 16630118	2006	Germany	135	IHC	IRS> 6	Survival curve	OS	2.09 (1.29-3.41)
Otsu H et al.	PMID: 27245623	2016	Japan	207	IHC	IRS≥6	Survival curve	OSRFS	1.77 (0.93-3.36)1.38 (0.95-2.02)
Jang YJ et al.	PMID: 16865274	2006	Korea	280	IHC	NR	Survival curve	OS	0.40 (0.17-0.91)
Chen ZW et al	CNKI	2009	China	80	IHC	Score≥2	NR	NR	NR
Sun YH et al.	CNKI	2014	China	60	IHC	IOD≥994	Survival curve	OS	2.25 (1.21-4.16)
Zou CG et al	CNKI	2009	China	49	IHC	Score≥2	NR	NR	NR
Lan B et al.	PMID: 17253180	2007	China	89	IHC	Score>6	Survival curve	OS	2.77 (1.09-4.73)
Zhang Q et al.	CNKI	2005	China	54	IHC	Positive cells≥6%	NR	NR	NR
Xia D et al.	CNKI	2014	China	80	IHC	SCORE>1	NR	NR	NR
Yao HL et al.	PMID: 21051833	2010	China	59	IHC	Positive cells≥6%	NR	NR	NR
TCGA	TCGA	2017	USA	415	NR	≥11.95	Multivariate analysis	OSRFS	1.25 (0.87-1.78)1.27 (0.93-1.75)
Kim HK et al.	GSE14208	2011	USA	123	GPL571	≥6.67	Univariate analysis	OSPFS	1.41 (0.97-2.06)1.47 (1.01-2.14)
Ooi CH et al.	GSE15459	2014	Switzerland	192	GPL570	≥8.38	Multivariate analysis	OS	0.95 (0.61-1.50)
Paik S et al.	GSE26253	2014	South Korea	432	GPL8432	≥9.07	Univariate analysis	RFS	0.68 (0.51-0.91)
Lee J et al (2).	GSE26901	2016	USA	109	GPL6947	≥7.18	NR	NR	NR
Lee J et al (1).	GSE28541	2012	USA	40	GPL13376	≥6.81	NR	NR	NR
Lei Z et al.	GSE34942	2014	Singapore	56	GPL570	≥5.52	Multivariate analysis	OS	1.96 (0.73-5.28)
Zhang X et al.	GSE63089	2014	China	45	GPL5175	≥6.79	NR	NR	NR
Cui J et al.	Oncomine	2011	USA	80	GPL5175	≥7.06	NR	NR	NR
Cho JY et al.	Oncomine	2011	USA	65	GPL6884	≥7.07	NR	NR	NR

NR: not reported; OS: overall survival; IHC: immunohistochemistry; RFS: recurrence-free survival; IRS: immunoreactivity scoring system; IOD: integrated optical density; PFS: progression-free survival.

### The expression level of PLK1 in GC via various databases

**Figure 2:**
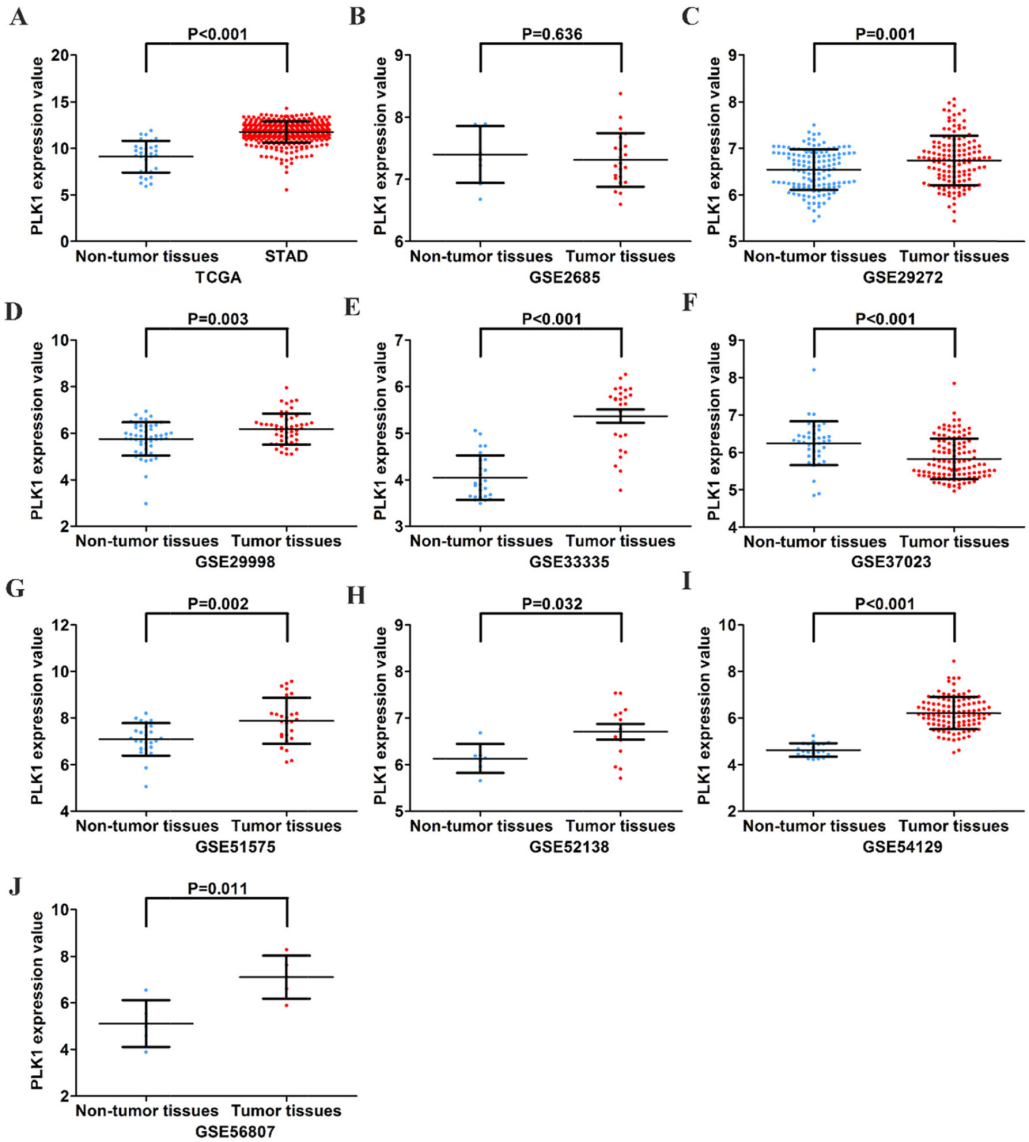
Different expression levels of PLK1 between gastric cancer and non-tumor gastric tissues based on 10 datasets **(A)** TCGA; **(B)** GSE2685; **(C)** GSE29272; **(D)** GSE29998; **(E)** GSE33335; **(F)** GSE37023; **(G)** GSE51575; **(H)** GSE52138; **(I)** GSE54129; **(J)** GSE56807.

**Figure 3:**
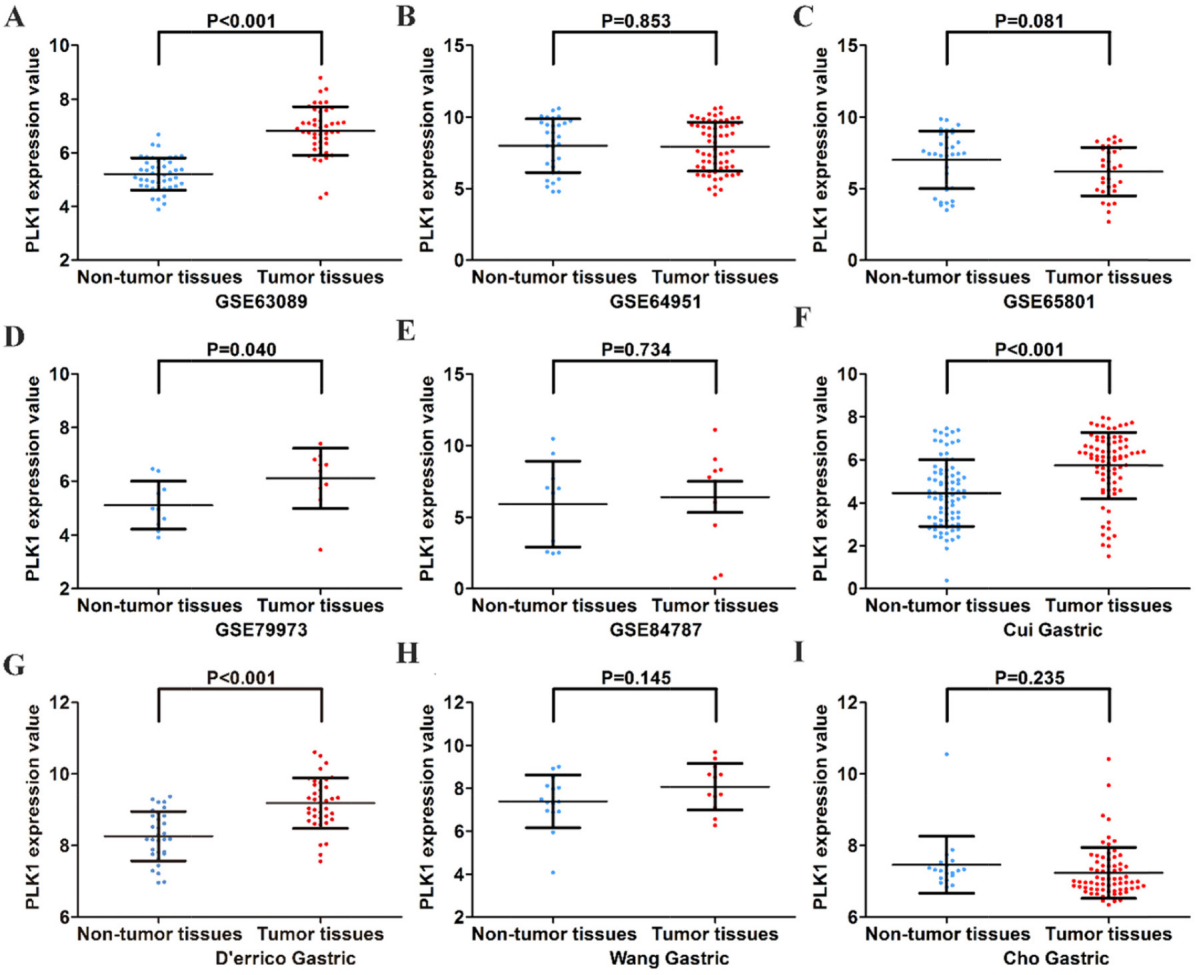
Different expression levels of PLK1 between gastric cancer and non-tumor gastric tissues based on another 9 datasets **(A)** GSE63089; **(B)** GSE64951; **(C)** GSE65801; **(D)** GSE79973; **(E)** GSE84787; **(F)** Cui Gastric; **(G)** D’errico Gastric; **(H)** Wang Gastric; **(I)** Cho Gastric.

**Figure 4:**
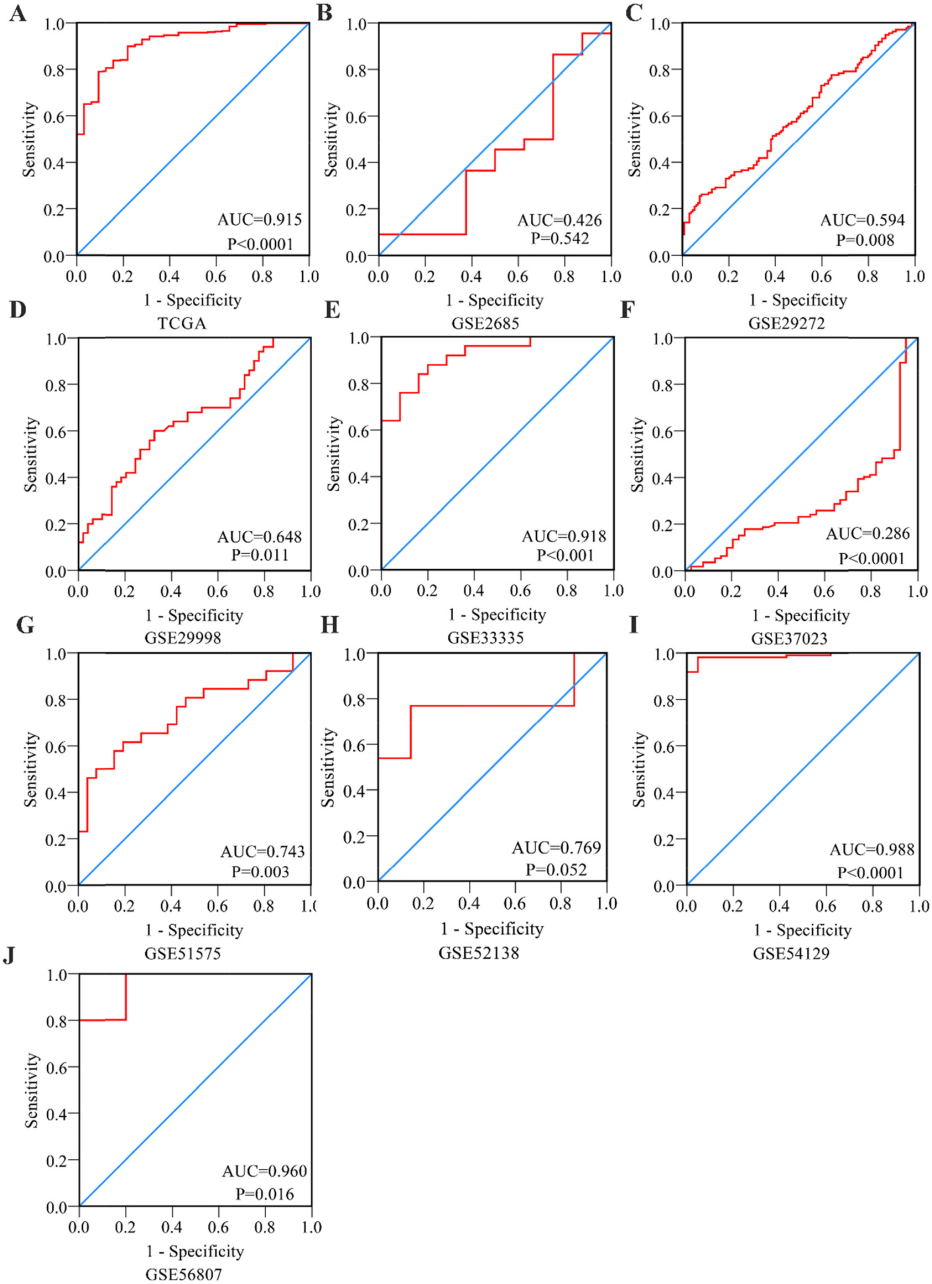
Receiver Operating Characteristic curves of PLK1 expression for the differentiation of gastric cancer from non-tumor tissues based on 10 datasets **(A)** TCGA; **(B)** GSE2685; **(C)** GSE29272; **(D)** GSE29998; **(E)** GSE33335; **(F)** GSE37023; **(G)** GSE51575; **(H)** GSE52138; **(I)** GSE54129; **(J)** GSE56807.

**Figure 5:**
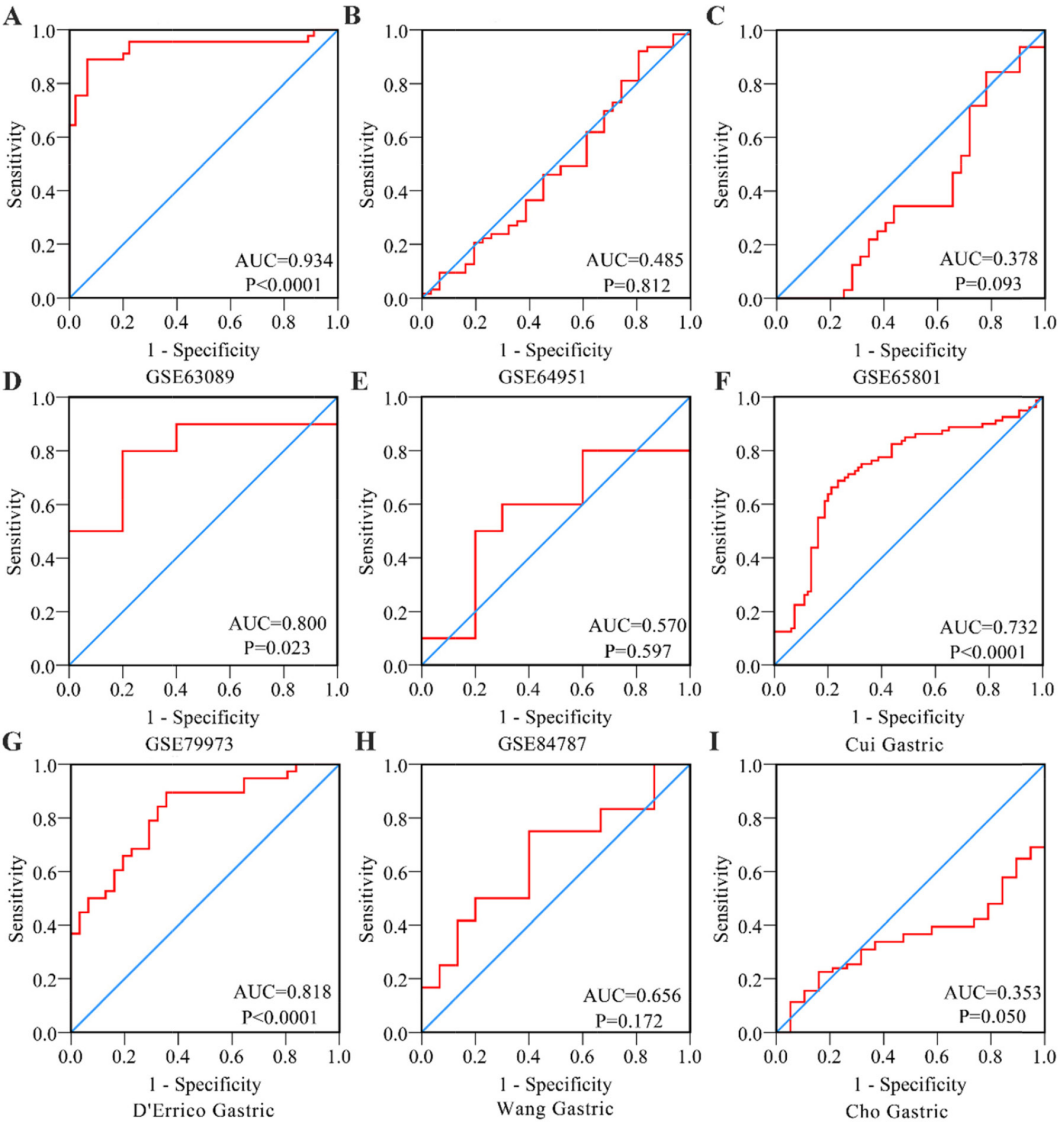
Receiver Operating Characteristic curves of PLK1 expression for the differentiation of gastric cancer from non-tumor tissues based on another 9 datasets **(A)** GSE63089; **(B)** GSE64951; **(C)** GSE65801; **(D)** GSE79973; **(E)** GSE84787; **(F)** Cui Gastric; **(G)** D’errico Gastric; **(H)** Wang Gastric; **(I)** Cho Gastric.

**Figure 6:**
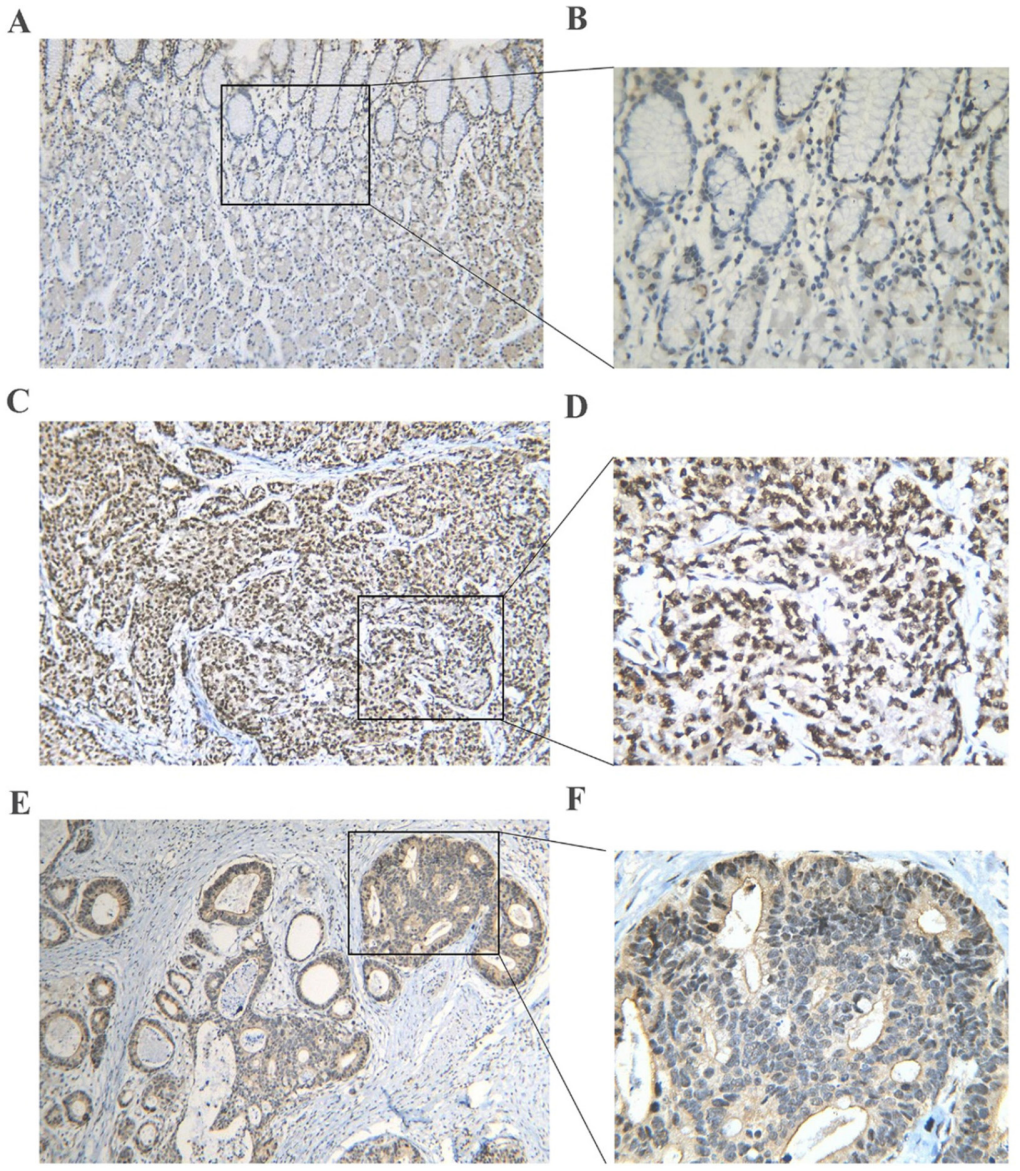
PLK1 protein expression in gastric cancer and non-tumor tissues assessed by immunohistochemistry **(A, B)** A normal gastric tissue showing low PLK1 expression. **(C, D)** A poorly differentiated tumor tissue showing high PLK1 expression. **(E, F)** A well differentiated tumor tissue showing high PLK1 expression. Magnification: ×100 (A, C, E) or ×400 (B, D, F).

First, by mining online databases (TCGA, GEO, and Oncomine databases), 19 datasets were obtained, which provided PLK1 mRNA expression values in GC tissues and adjacent non-tumor tissues. We investigated the expression pattern of PLK1 in gastric cancer based on each independent dataset. In Figure 2, 3, 4 and 5, the PLK1 expression pattern of each data set was displayed in the form of scatter plots and receiver operating characteristic (ROC) diagrams. Then, we detected the expression of PLK1 protein in 43 pairs of GC tissues by IHC staining. Among these GC samples, the immunoreactivity score (IRS) indicated that PLK1 protein expression was significantly higher in the 43 GC samples (9.42±3.06) than in adjacent non-tumor tissues (7.02±3.17, P=0.001, Figure 6).

### Verification of PLK1 up-regulation in GC via a meta-analysis

**Figure 7:**
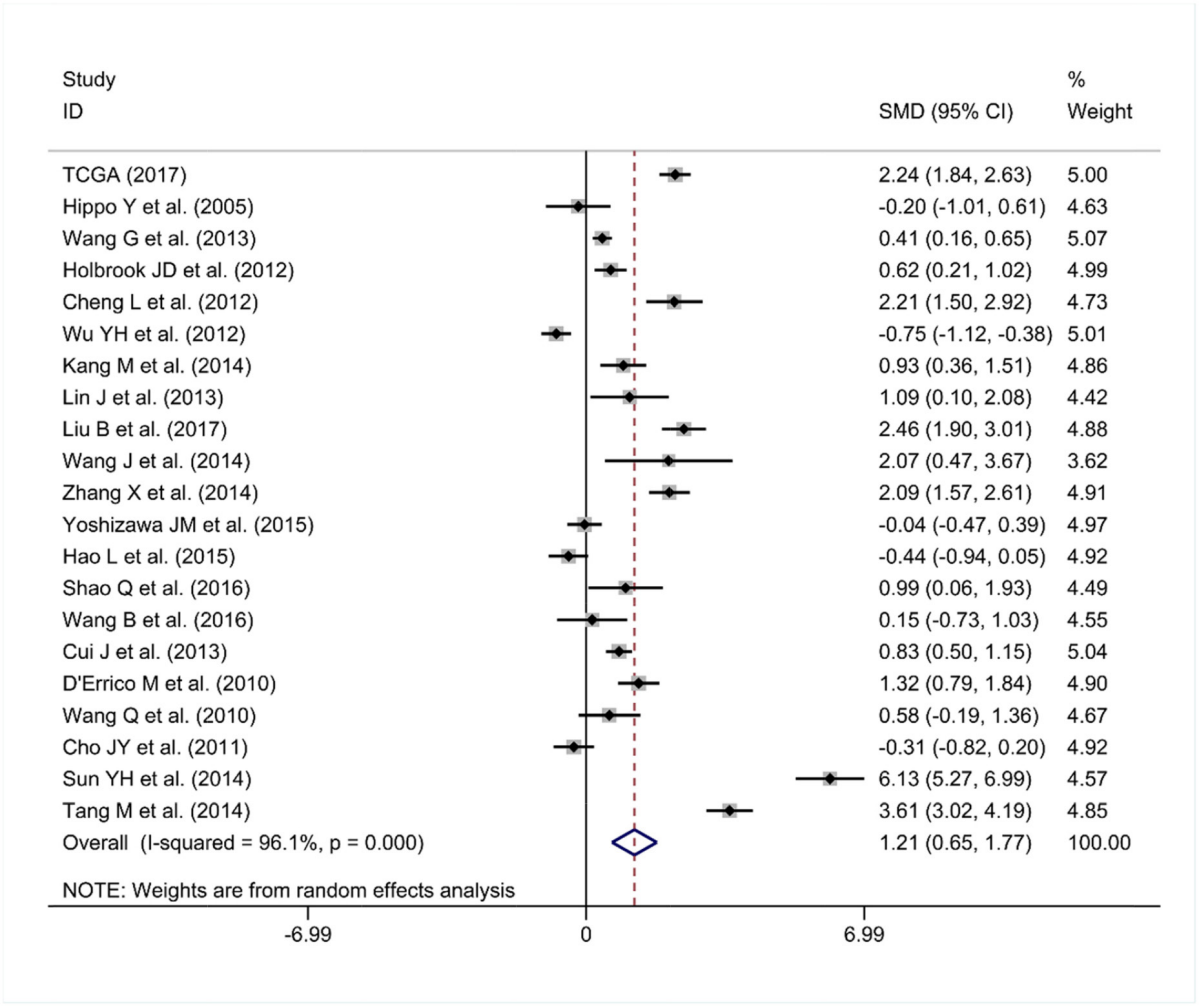
Forest plot for evaluating PLK1 mRNA expression between gastric cancer and non-tumor tissues

**Figure 8:**
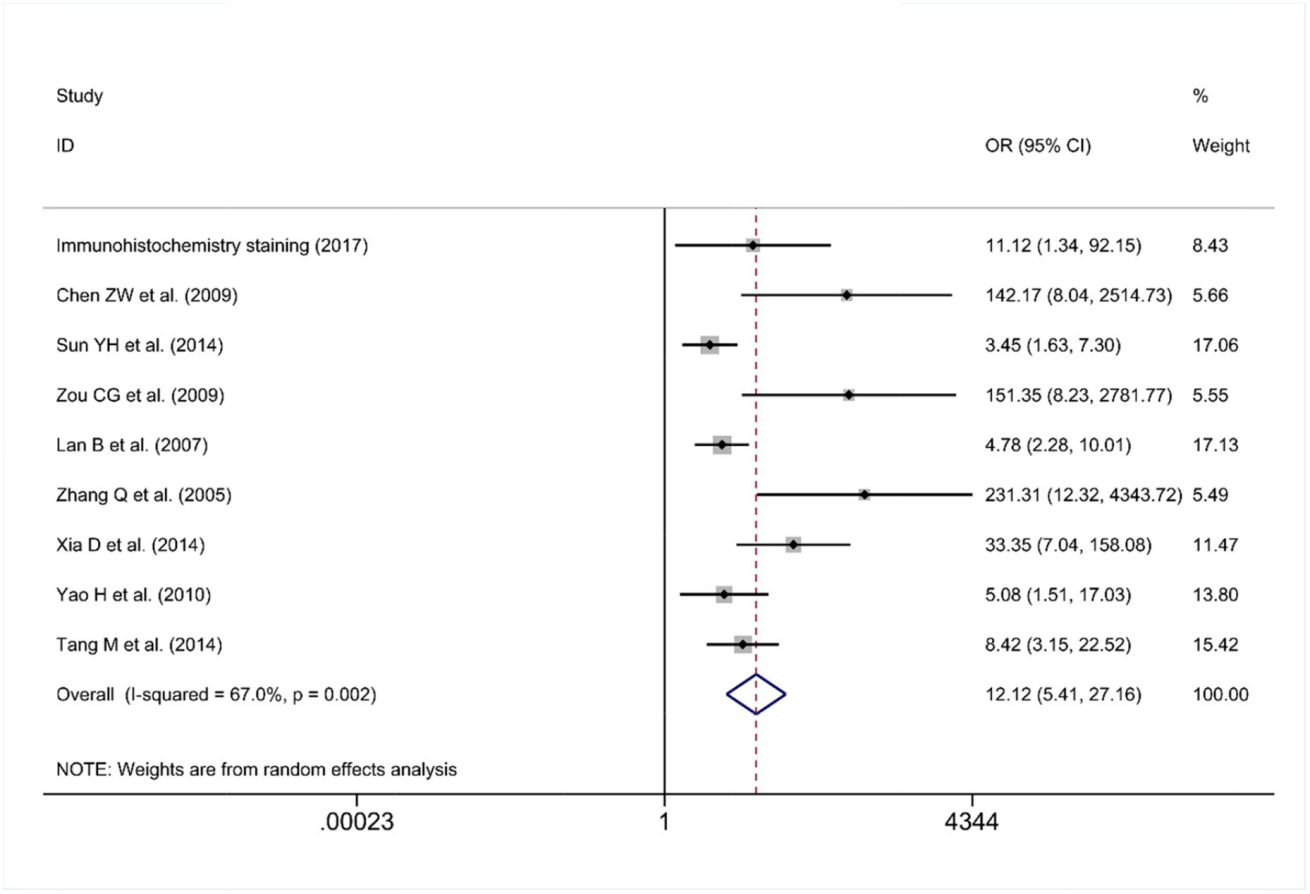
Forest plot for the different PLK1 protein expression levels between gastric cancer and non-tumor tissues

As some individual studies were too small to yield a valid conclusion, we integrated all of the data. A random-effects model was selected, as apparent heterogeneity existed among the 21 studies, which were listed in Table 1 (I^2^ = 96.1%, P<0.001; Figure 7). The pooled standard mean deviation (SMD) of PLK1 mRNA was 1.21 (95% CI: 0.65-1.77, P< 0.001; Figure 7), which suggested that PLK1 mRNA was remarkably up-regulated in GC. Additionally, the pooled odds ratio (OR) calculated based on 7 articles and our IHC staining results revealed that PLK1 protein expression was also up-regulated in GC compared to normal gastric tissue (OR=12.12, 95% CI: 5.41-27.16, P<0.001, random effect model; Figure 8) for heterogeneity (I^2^ =67%, P=0.002).

**Figure 9:**
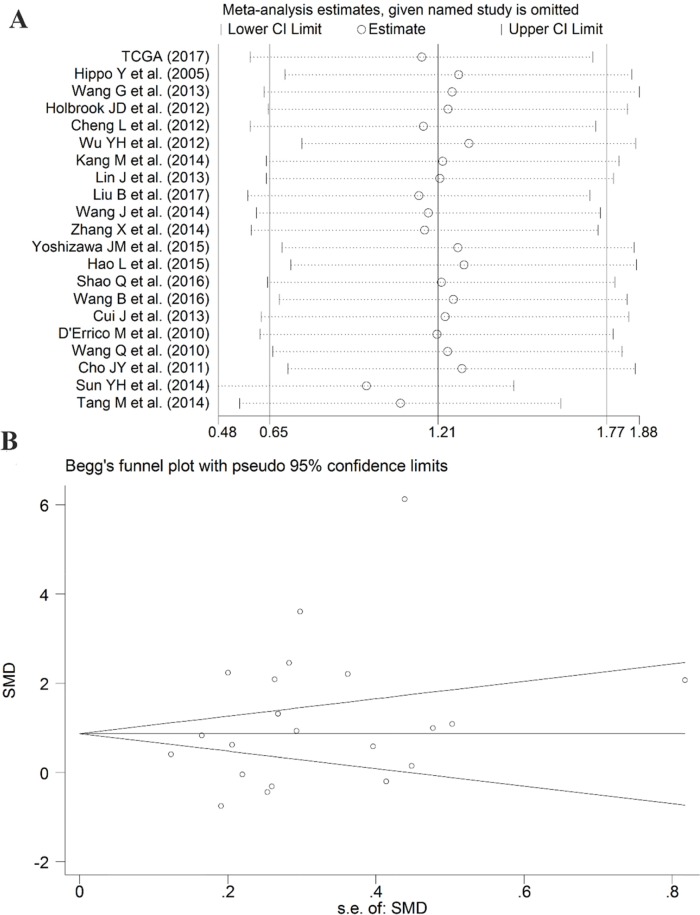
Results of the sensitivity analysis and Begg's funnel plot of PLK1 expression **(A)** Sensitivity analysis of standard mean deviation (random effects model) based on stepwise omitting one study at a time. **(B)** Analysis of the detection of publication bias in the meta-analysis of studies assessing the expression pattern of PLK1 in gastric cancer.

Sensitivity analysis suggested that the pooled SMD was stable (Figure 9A). Furthermore, Begg's funnel plot and Egger's test were carried out to visualize the publication bias. Begg's regression plot showed no potential publication bias (P= 0.139, Figure 9B). Equally, Egger's test indicated that no publication bias was found for the PLK1 overexpression in GC (P= 0.134). In summary, these current results confirmed the overexpression of PLK1 in GC.

**Figure 10:**
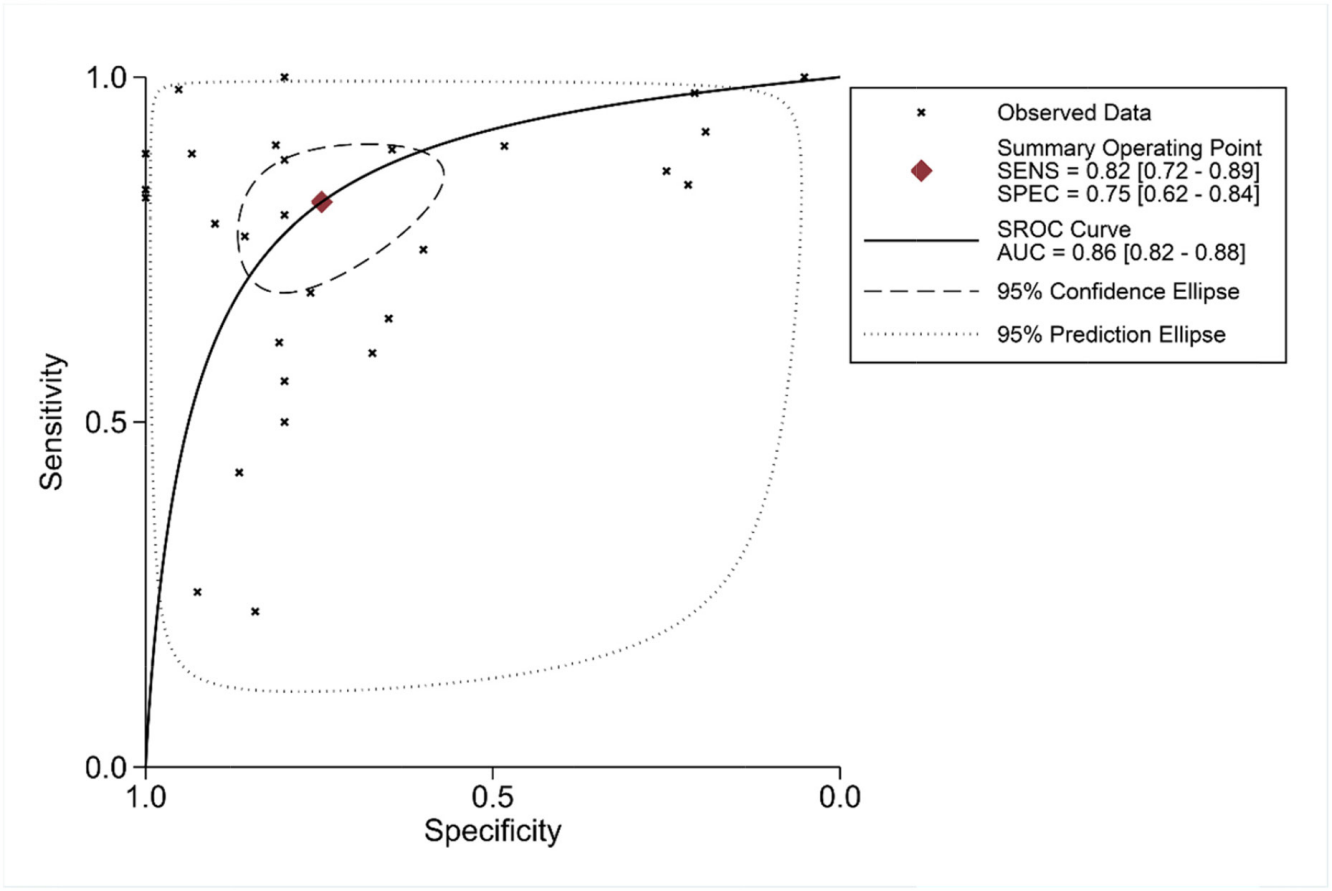
Summary receiver operating characteristic curve of the distinguishing capability of PLK1 for cancer from non-cancerous tissues

To further identify the capability of PLK1 in discriminating cancer from non-cancerous gastric tissues, we generated a summary receiver operating characteristic (SROC) curve and then calculated the area under the curve (AUC). A total of 28 groups of data obtained from TCGA, Oncomine, GEO, publications and IHC staining were summarized. The overall AUC of PLK1 in GC was 0.86 (95% CI: 0.82-0.88), with a sensitivity and specificity of 0.82 (95% CI: 0.72-0.89) and 0.75 (95% CI: 0.62-0.84), respectively (Figure 10).

### PLK1 overexpression and clinicopathological features in GC patients

For clinical parameters, we performed integrative analyses and revealed that PLK1 overexpression in GC patients was associated with several clinicopathological parameters. As shown in Table 3, the increased expression of PLK1 was positively associated with lymph node metastasis (OR: 1.78, 95% CI: 1.13–2.80, P=0.013, random effect model) and advanced TNM stage (OR: 1.48, 95% CI: 1.02–2.15, P=0.038, random effect model). No significant relationships were observed between PLK1 expression and age (OR=1.19, 95% CI: 0.77-1.84, P=0.437, random effect model), gender (OR=0.87, 95% CI: 0.71-1.07, P=0.191, fixed effect model), histology grade (OR=1.07, 95% CI: 0.66-1.73, P=0.783, random effect model), depth of tumor invasion (OR=1.70, 95% CI: 0.97-2.98, P=0.061, random effect model), or metastasis (OR=1.37, 95% CI: 0.79-2.37, P=0.209, fixed effect model).

**Table 3:** The relationships between PLK1 overexpression and clinicopathological significance in gastric cancer patients

Clinicopathological features	Studies (n)	Patients (n)	Meta-analysis model	OR (95% CI)	P	Heterogeneity	
						I^2^(%)	p
Lymph node metastasis	12	1395	random effects model	1.78(1.13–2.80)	0.013	64.2	0.001
TNM stage	15	1937	random effects model	1.48(1.02-2.15)	0.038	63.6	<0.001
Age	8	1006	random effects model	1.19(0.77-1.84)	0.437	53.2	0.037
Gender	15	1915	fixed effects model	0.87(0.71-1.07)	0.191	23.2	0.197
Tumor histology grade	13	1447	random effects model	1.07(0.66-1.73)	0.783	64.9	0.001
Depth of tumor invasion	12	1405	random effects model	1.70(0.97-2.98)	0.061	78.6	<0.001
Metastasis	5	667	fixed effects model	1.37(0.79-2.37)	0.209	31.9	0.209

TNM: tumor node metastasis.

### Evaluation of the prognostic value of PLK1 in GC

**Figure 11:**
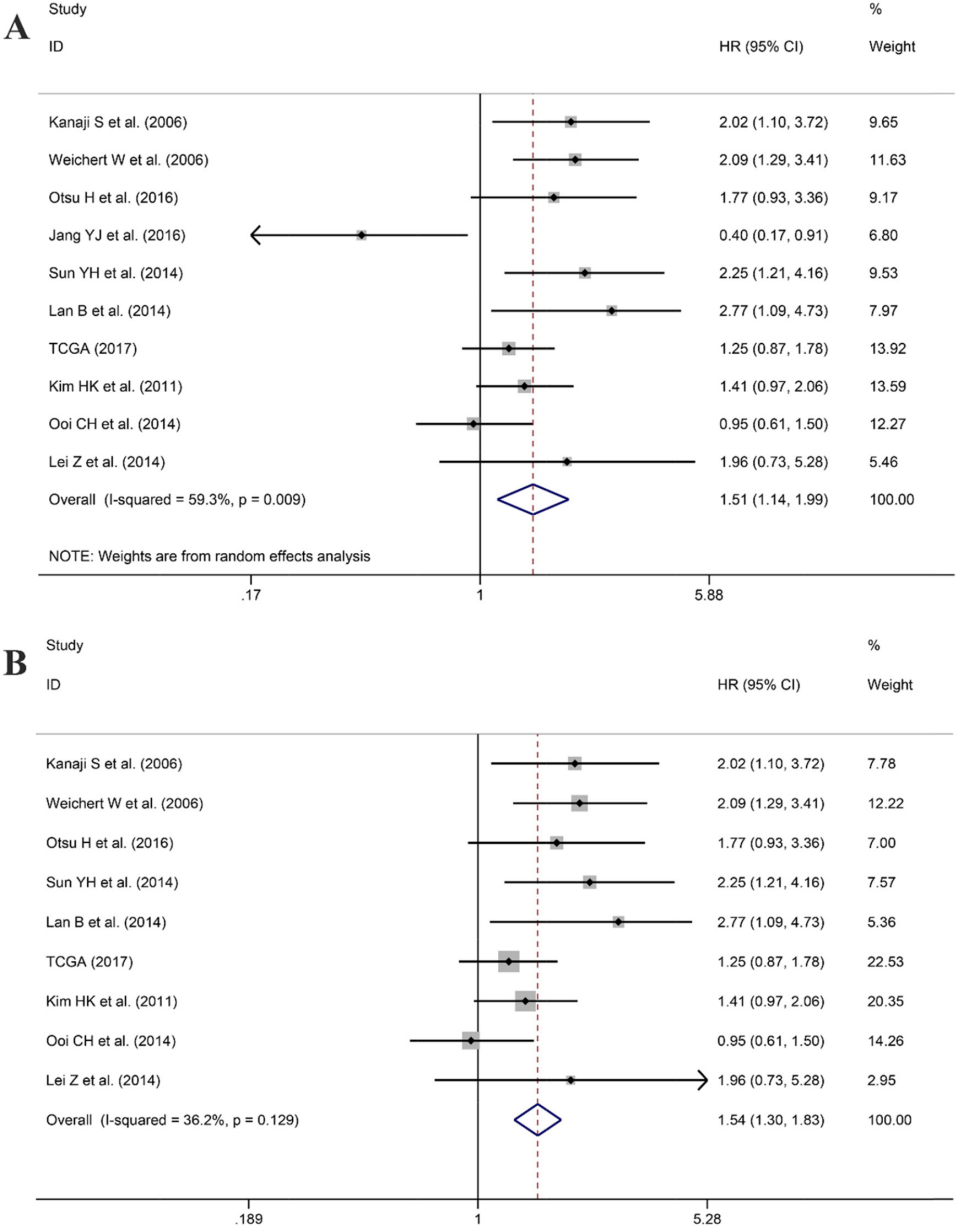
Forest plot for the association between PLK1 expression and overall survival (OS) **(A)** Correlation between OS and PLK1 expression based on all studies included. **(B)** Correlation between OS and PLK1 expression after the study of Jang et al. was removed.

**Figure 12:**
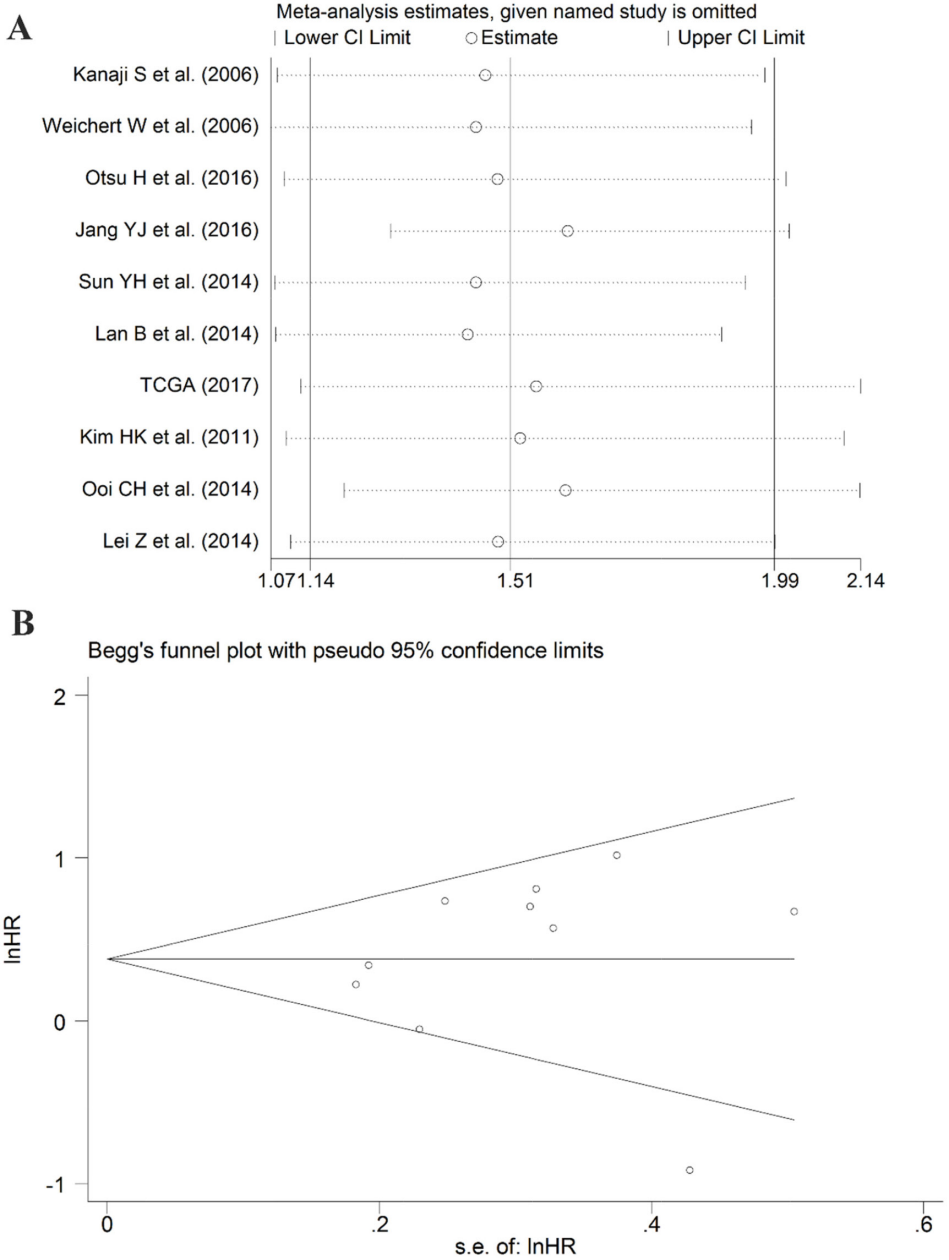
Results of the sensitivity analysis and publication bias **(A)** Sensitivity analysis of the hazard ratio (random effects model), calculated by sequentially each study removal. **(B)** The funnel plot detecting the potential publication bias among the included records.

After screening, ten studies assessed the correlation between overexpressed PLK1 and overall survival (OS), with three studies containing information regarding the association between up-regulated PLK1 and recurrence free survival (RFS). Additionally, one study had sufficient information to calculate the relationship between high PLK1 expression and progression-free survival (PFS). It indicated that PLK1 overexpression was markedly correlated with worse OS (hazard ratio (HR): 1.51, 95% CI: 1.14–1.99, P=0.004; Figure 11A) with significant heterogeneity (I^2^ =59.3%, P=0.009) when employing the random-effect model. Furthermore, a sensitivity analysis was performed, and the result implied the pooled HR was stable (Figure 12A). As shown in Figure 12B, Begg's regression plot revealed no evidence of potential publication bias (P = 0.641). Additionally, Egger's test indicated no statistically significant publication bias was found for the positive PLK1 expression on OS (P = 0.721). To minimize heterogeneity, we omitted the article written by Jang YJ, as the overexpression of PLK1 was significantly correlated with a shorter OS (HR =1.54, 95% CI: 1.30–1.83, P<0.001; Figure 11B) with no heterogeneity (I^2^= 36.2%, P =0.129). The overexpression of PLK1 clearly led to an inferior PFS (HR: 1.47, 95% CI: 1.01-2.14). However, the result had low credibility as only 1 study provided data. Additionally, the RFS between high and low expression levels of PLK1 did not differ significantly (HR: 1.05, 95% CI: 0.67–1.66, P=0.834).

### Relevant genes of PLK1 and gene-annotation enrichment analysis

**Figure 13:**
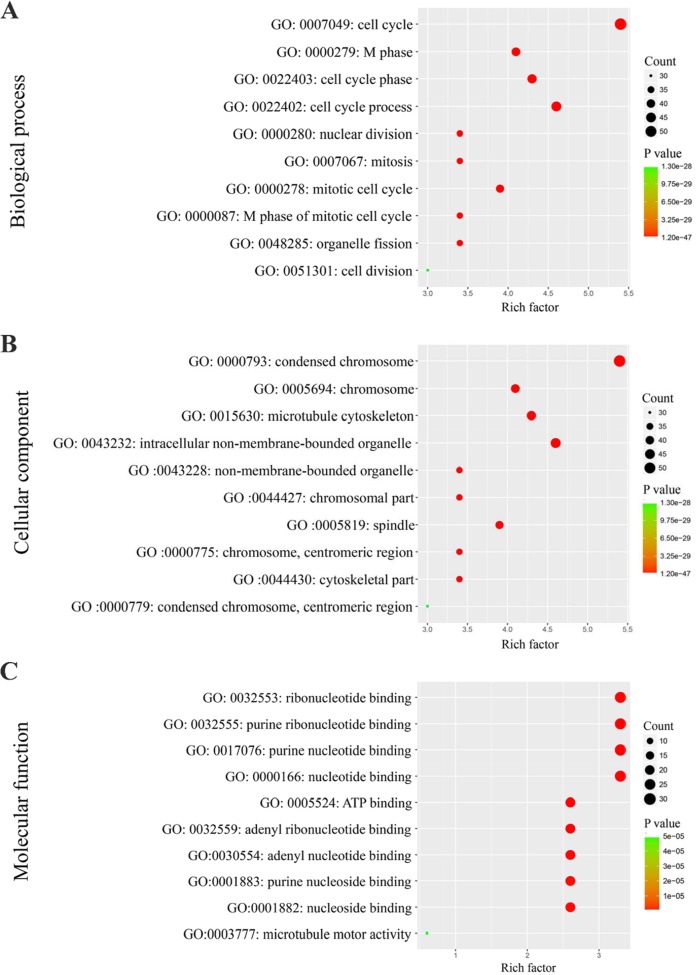
Gene Ontology analysis of the genes relevant to PLK1 in GC **(A)** Biological process; **(B)** Cellular component; **(C)** Molecular function.

**Figure 14:**
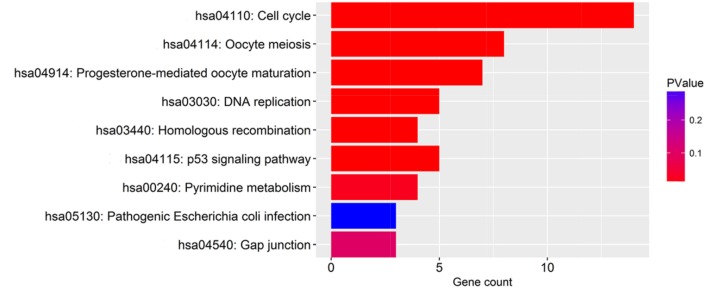
The significantly enriched annotation of the Kyoto Encyclopedia of Genes and Genomes pathway analysis of the genes relevant to PLK1 in GC

The 100 genes most relevant to PLK1 in GC were obtained using Gene Expression Profiling Interactive Analysis (GEPIA) as described. Determining the biological value of these genes could provide important information on the mechanism of PLK1 in GC. These 100 relevant genes were input into DAVID to perform GO and Kyoto Encyclopedia of Genes and Genomes (KEGG) pathway analysis. As shown in Figure 13, GO analyses were implemented in three categories, including biological process (BP), cellular component (CC) and molecular function (MF). For BP, the most notably enriched functional terms were cell cycle, M phase, and cell cycle phase (P<0.001). Regarding CC, genes markedly assembled at condensed chromosome, chromosome and microtubule cytoskeleton (P<0.001). On the basis of MF, genes prominently accumulated in ribonucleotide binding, purine ribonucleotide binding and purine nucleotide binding (P<0.001). Additionally, these genes in the KEGG enrichment analysis were shown to be particularly related to the cell cycle (P<0.001, Figure 14).

### Protein–protein interactions (PPI) network construction and the hub genes identification

**Figure 15:**
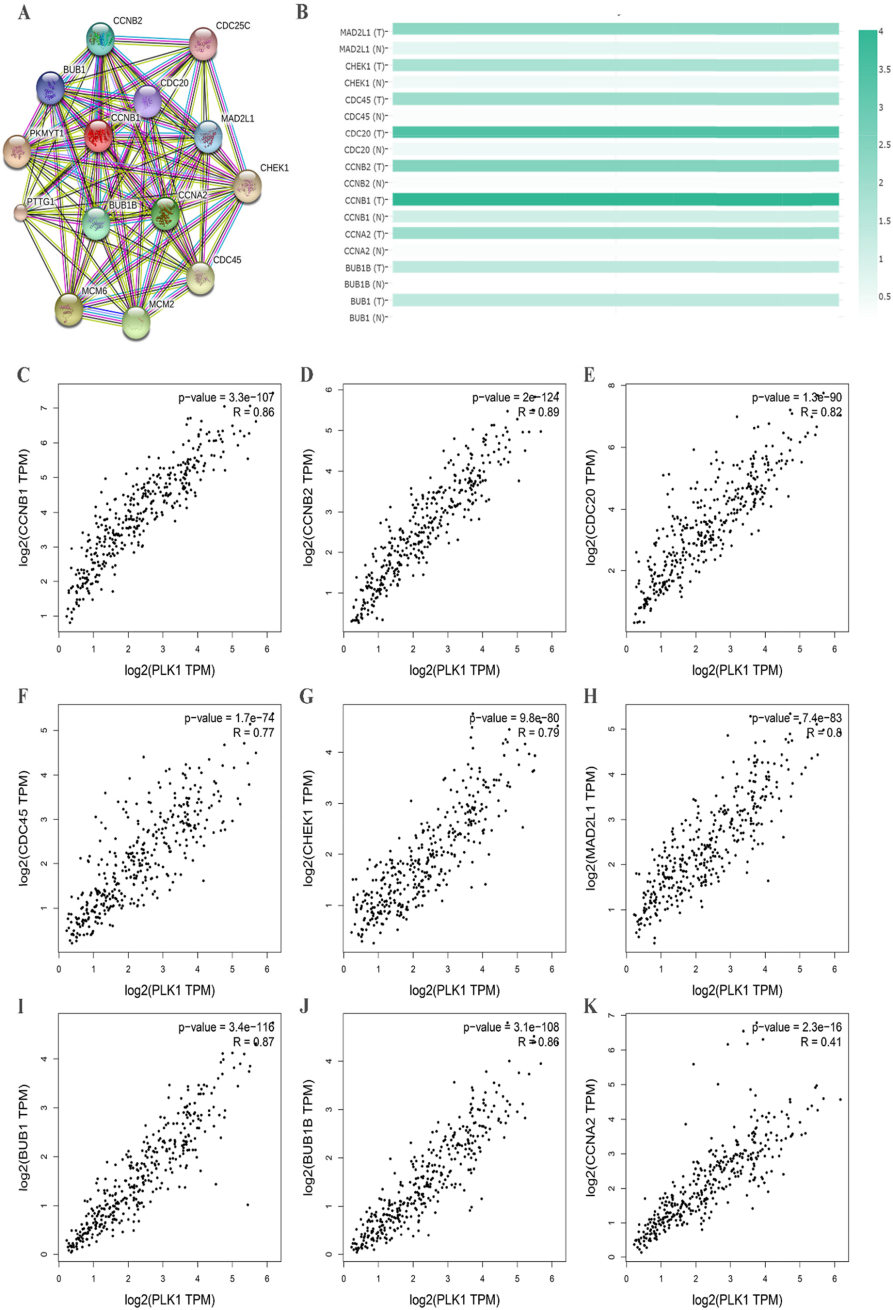
Protein-protein interaction (PPI) network of several related genes enriched in cell cycle pathway **(A)** PPI network was drawn using STRING online tool. **(B)** Plots of nine hub genes drawn by GEPIA online tool. The density of color in each block represented the median expression value of a gene. **(C-K)** Scatter plot showed the correlation between the PLK1 expression and the nine hub genes signature.

To further insight into the molecular mechanism of PLK1, we used a functional protein network database STRING to construct the PPI network. Through the constructed PPI network, nine hub genes of PLK1 were obtained (Figure 15A). These genes were MAD2L1, CHEK1, CDC45, CDC20, CCNB2, CCNB1, CCNA2, BUB1B and BUB1. Then, we also provided expression matrix plots of these hub genes (Figure 15B). Furthermore, the correlations between PLK1 and hub genes were calculated (Figure 15C-15K). These nine genes were positively correlated with PLK1.

### Identification of PLK1-mediated molecular functions in GC by GSEA

**Figure 16:**
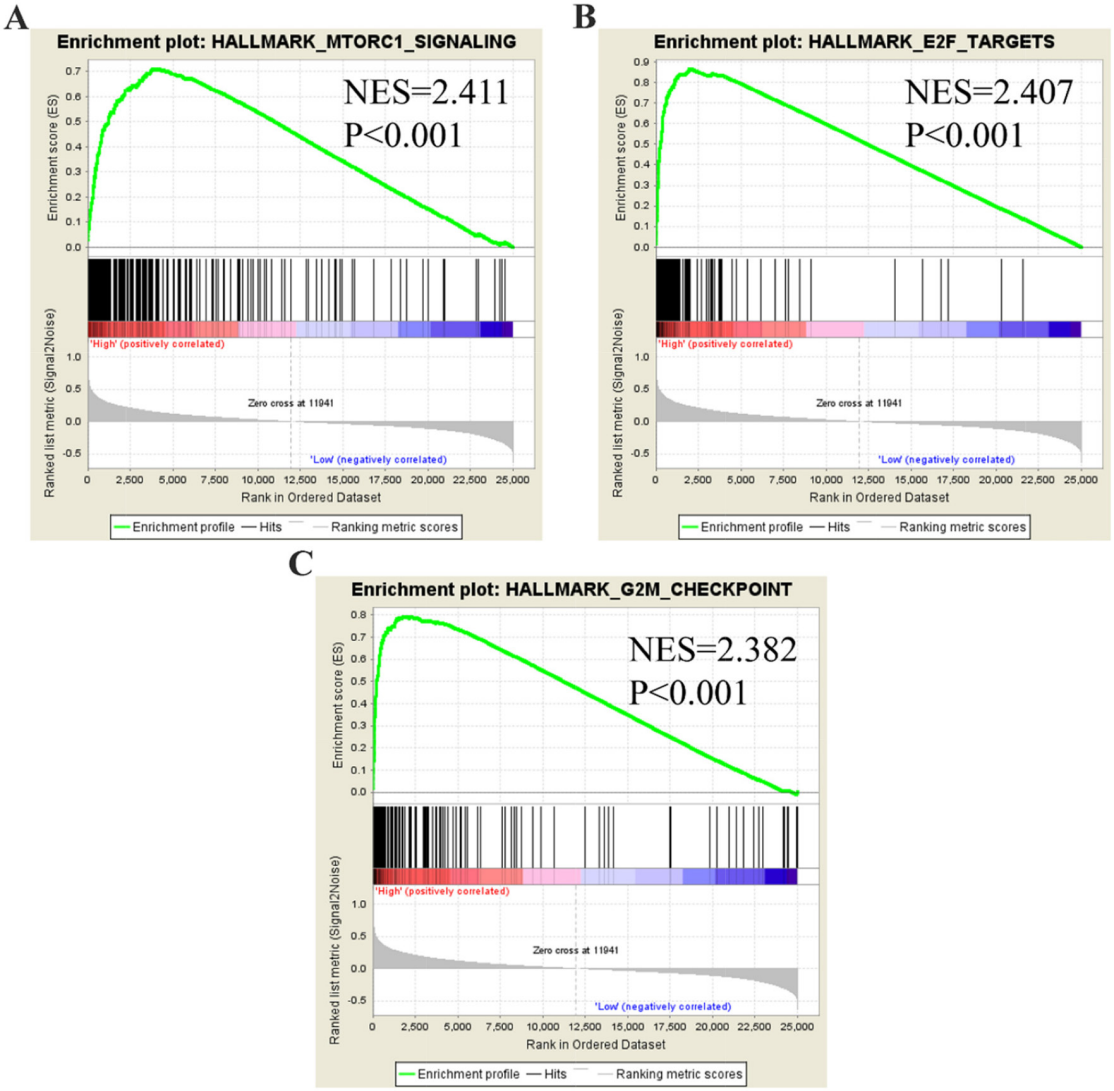
GSEA plot showing that PLK1 expression positively correlated with the three most significant enrichment sets **(A)** MTORC1 signaling; **(B)** E2F targets; **(C)** G2M checkpoints.

To identify molecular functions of PLK1 in GC in-depth, we performed Gene Set Enrichment Analysis (GSEA) using TCGA data. Among all of the predefined hallmark gene sets, a total of 9 items were most commonly associated with PLK1 overexpression in the TCGA cohort (FDR q-value<0.01), including MTORC1 signaling, E2F targets and the G2M checkpoint, etc. (Figure 16), suggesting that PLK1 may be deeply engaged in GC tumorigenesis and progression through several cancer-associated signaling pathways.

## DISCUSSION

Identification of risk factors and accurate prognostic prediction in GC patients are critical to selecting appropriate therapies and guiding clinical follow-up [28–30]. Unfortunately, accurately identifying the risks in individual patients is relatively difficult [31–33]. Although accumulating advances have been achieved to further understand the clinical value of PLK1 in GC, the conclusions have been controversial. Therefore, a comprehensive view of PLK1 in GC is needed. Taking advantage of the vast publicly available databases, we sought to unravel a crucial function of PLK1 in GC via excavating the Oncomine, TCGA, and GEO databases, published literature and IHC staining results in our hospital. Here, we first gathered all-inclusive data to confirm that PLK1 is markedly up-regulated in GC patients. Furthermore, we observed significant differences between PLK1 high and low expression levels regarding tumor pathological stage. More importantly, GC patients with PLK1 overexpression had inferior survival outcome and a significantly increased risk of lymph node metastasis. Through the bioinformatics analysis, we firmly verified that PLK1 played a vital part in the regulation of the cell cycle and contributed to cancer-related signaling pathways, thus contributing to the stepwise tumorigenesis and progression in GC. Based on the above evidence, cautious monitoring of PLK1 could be clinically useful for decision-making in GC.

The first intention of the study was to clarify the expression pattern of PLK1 in GC. As expected, a pooled SMD reached 1.21 (95% CI: 0.65-1.77, P<0.001) presented by the random-effects model and no publication bias was observed. Similar to PLK1 mRNA, the pooled OR also revealed remarkably increased expression of PLK1 protein in GC, in comparison to normal gastric tissue. Furthermore, a robust ability of PLK1 to distinguish cancer from non-cancerous gastric tissues was observed via ROC (AUC: 0.86; 95% CI: 0.82–0.88, P<0.001). These demonstrated that the expression levels of PLK1 in GC tissues were significantly overexpressed. As a consequence, PLK1 might act as a cancer promoting factor and participate in GC tumorigenesis.

To further evaluate the clinical relevance and prognostic value of PLK1 in GC patients, we analyzed, for the first time, the remarkably increased expression of PLK1 as an unfavorable prognostic factor for OS in GC patients using a meta-analysis. The prognostic value of PLK1 in cancers is currently high profile. Zhang et al performed a meta-analysis using eleven eligible articles and demonstrated that an increased expression of PLK1 indicated a higher risk of worse survival in breast cancer patients [12]. Liu et al compared the OS time between higher PLK1 and lower PLK1 groups in 25 cancer types based on the data provided by TCGA. The study revealed that cases with higher PLK1 suffered remarkably worse OS as compared to those with lower PLK1 in 10 cancer types: adrenocortical carcinoma, bladder urothelial carcinoma, breast invasive carcinoma, kidney renal clear cell carcinoma, kidney renal papillary cell carcinoma, brain lower-grade glioma, lung adenocarcinoma, pancreatic adenocarcinoma, skin cutaneous melanoma, and uterine corpus endometrial carcinoma [34]. For GC, the results from our study fully proved that PLK1 overexpression may be an indispensable biomarker for the accurate assessment of prognosis. Additionally, this meta-analysis documented that elevated PLK1 was closely linked with advanced TNM stage and lymph node metastasis. Metastasis is a major death cause in cancers and complicates treatment opportunities [35]. Hence, an in-depth comprehension of the molecular mechanism of PLK1 in tumor metastasis and inferior prognosis may pave the way toward new prognostic models.

The mechanisms responsible for PLK1 in GC are still not clear. We collected 100 genes that had a similar expression pattern to PLK1 in GC and conducted GO and KEGG pathway enrichment analysis. GO enrichment analysis indicated the possible functions of PLK1 in GC, and the results from three GO terms and KEGG pathway analysis determined that PLK1 was mainly involved in the cell cycle, which might be the mechanism by which PLK1 exerts its versatile and critical biological function in GC. Consistent with the results from GO and KEGG analysis, PLK1, as a master controller of the cell cycle, has been widely recognized [36, 37]. Several studies have identified that PLK1 could impair the apoptosis, and enhance mitosis, cell growth and metastasis potential of GC cells. For example, in one of the GC cell lines, SNU-638, Jang YJ [19] found that depletion of PLK1 inhibited cell proliferation and caused apoptosis. Interestingly, Chen XH [38] also confirmed similar results by blockage of PLK1 expression in GC cells MKN45. To further probe into the molecular mechanism of PLK1, we obtained 9 hub genes by using PPI analysis. These hub genes were MAD2L1, CHEK1, CDC45, CDC20, CCNB2, CCNB1, CCNA2, BUB1B and BUB1, which were all upregulated in GC significantly and were positively related to PLK1. Hence, it could be speculated that PLK1 regulates cell cycle pathways by co-operating with these nine genes, and thereby facilitates the carcinogenesis and development of GC. Based on these findings, PLK1 is regarded as a key player in orchestrating the cell cycle, leading to the progression of GC.

In addition to GO and KEGG analysis, the results from GSEA suggested that MTORC1 signaling was most closely related with elevated PLK1 expression in GC patients. Today, most studies on PLK1 and MTORC1 signaling have been separate, and little is known about the crosstalk between them. Recently, Ruf et al proposed that PLK1 inhibited MTORC1 and thereby positively contributed to autophagy in HeLa cells [39]. The role of autophagy in cancer is one that has been highly researched but is still deeply mysterious. Cai et al [16] demonstrated that PLK1 drove gastric tumor epithelial-mesenchymal transition (EMT) via targeting AKT signaling. AKT is also a key part of the MTORC1 signaling pathway and is closely related to metastasis, proliferation and invasion processes of gastric cancer [40–42]. All of the findings above lead to the opinion that PLK1 may be involved in autophagy in GC.

Molecularly targeted therapy and prognosis assessment have opened up new avenues for clinical cancer treatment. For molecular therapy, several preclinical studies with PLK1 inhibitors are underway [43–45]. Thus, we speculated that the combinatorial targeting of MTORC1 or AKT and PLK1 may be more meaningful and efficient for clinicopathological and prognostic estimation. Although some preclinical studies have been terminated due to side effects, looking for efficient and specific PLK1 molecular inhibitors will be the next priority for the clinical treatment of tumors. This truly pleiotropic kinase, assuredly, will continue to fascinate in the future.

However, the results should be interpreted cautiously due to some limitations that may have influenced the reliability of our conclusions. First, PLK1 expression was uniformly analyzed by various detection methods and diverse RNA detection platforms. This may be caused by the lack of standardization, which may result in inaccurate results. Therefore, a large multicenter study is needed to determine the most suitable detection method of PLK1. Consequently, the random-effects model was selected to reduce the impact of the heterogeneity on our results. Second, as some studies did not provide accurate original survival data, some of the survival data were indirectly extracted from Kaplan-Meier curves by Engauge Digitizer. Accordingly, the corresponding HR and 95% CI may lack credibility. Third, specific signaling pathways regulated by PLK1 in GC still must be validated using *in vitro* or *in vivo* experiments.

Taken together, the study proposes that PLK1 is a valid prognostic marker and will play a significant role in future clinical trials. Future, more high-quality and big population studies are desirable to update this analysis.

## MATERIALS AND METHODS

### Data mining

Microarray or RNA-seq datasets, which were available for PLK1 expression pattern and relevant clinical information appraisal in GC, were downloaded and extracted from TCGA data portal (https://cancergenome.nih.gov/), GEO (https://www.ncbi.nlm.nih.gov/geo/) and Oncomine (https://www.oncomine.org/resource/main.html). In addition, a computer-aided systematic literature search was also performed in electronic databases PubMed, Embase, MEDLINE, Web of Science, Cochrane library, China National Knowledge Infrastructure (CNKI), and Wanfang, terminating in June 2017. The following keywords were used: (“polo-like kinase 1” OR STPK13 OR PLK1) AND (malignan^*^ OR cancer OR tumor OR tumour OR neoplas^*^ OR carcinoma) AND (gastric OR stomach). Cited references from identified primary studies or reviews were also manually scanned to avoid missing extra related studies.

### Data selection principles

For microarray and RNA-seq datasets, eligible records were included if they fulfilled all of the principles as follows: (1) there was a proven diagnosis of GC in humans; (2) data included expression profiling data of PLK1 in GC and could be used for analysis; (3) the number of samples included in each record containing PLK1 expression value in tumor and non-tumor was not less than 10; and (4) the records used for analyzing the clinicopathological significance of PLK1 included more than 30 samples.

All eligible published studies were required to match the criteria listed below: (1) diagnosis of GC was confirmed pathologically; (2) the clinical value of the PLK1 in GC patients was reported; (3) the study was original; (4) an OR with 95% confidence interval (CI) of clinicopathological parameter or a HR and their 95% confidence intervals (CIs) could be obtained from the article directly or estimated based on the information in the paper sufficiently; and (5) studies were published as a full-text paper in English or Chinese, although no language restrictions were imposed initially.

### Data extraction

Two investigators reviewed the included datasets and publications and extracted the relevant data for the study independently. Ambiguous or unclear details were determined through discussion with the third investigator. For datasets and literature relating to the expression level of PLK1, the following parameters were retrieved: first author, year of publication, country, data source, test method or platform, expression values of PLK1, sample size in both cancer and control groups and AUC value. To further confirm its dysregulated expression, true positive (TP), false positive (FP), false negative (FN) and true negative (TN) were also mined directly or assessed by the ROC curve analysis (data not shown).

For records on the prognostic role or clinicopathological significance of PLK1, the extracted characteristics comprised first author, data source, year of publication, the region of publication, sample size, test method or detection platform of PLK1 expression, cut-off values, type of survival data and HR with its 95% CI. Because multivariate analysis takes multi-parameterized associations into consideration, it would be more accurate [46]. Therefore, when univariate and multivariate analysis were presented simultaneously, we choose the latter. HRs and their 95% CIs were extracted from the original studies directly if provided. If HRs were not reported in the original study directly, the data were estimated from the Kaplan-Meier (K-M) survival curves by the software Engauge Digitizer version 4.1 (http://markummitchell.github.io/engauge-digitizer/) [47]. Additionally, we also calculated HRs using univariate and multivariate Cox analyses based on the expression level of PLK1 and follow-up data provided by datasets. ORs with their 95% CIs were assessed and the correlation between high expression of PLK1 and general clinicopathological parameters, including depth of tumor invasion (T1+T2 vs. T3+T4), gender (female vs. male), age (<60 vs.≥60), lymph node metastasis (negative vs. positive), histology grade (G1+G2 vs. G3+G4), tumor TNM stage (I+II vs. III+IV) and metastasis (negative vs. positive) were evaluated. For those datasets that provided PLK1 expression value and clinical information, we extracted the requisite data by dividing patients into high PLK1 expression and low PLK1 expression groups based on selecting the median value of PLK1 as a cut-off value.

### Immunohistochemistry staining and evaluation

The expression level of PLK1 protein was detected by IHC staining based on 43 GC patients and their adjacent normal gastric tissues, which were obtained from the First Affiliated Hospital of Guangxi Medical University, People's Republic of China from January 2016 to April 2017. Formalin-fixed paraffin-embedded tissue samples were prepared into 4-μm-thick tissue sections. Then, the sections were dewaxed. Antigen retrieval was performed by pressure cooking at 95°C for 1 h. The samples were incubated with the first PLK1 rabbit polyclonal antibody diluted 1:500 (Abcam) at 37°C for 1 h. The rest procedure of immunohistochemistry was performed as the manufacturer's instruction introduced, and the final results were determined by two pathologists independently (Yi-wu Dang and Gang Chen). To investigate the regional differences in staining, the IRS was applied. Under light microscopy, 10 typical high-power visual fields were observed at random. The following two parameters were evaluated including the staining intensity and percentage of cells being stained in each sample to calculate the final IRS. The staining intensity was recorded as 0 if no staining was observed, 1 for weak staining, 2 for moderate staining and 3 for strong staining. Meanwhile, the percentage of cells stained was recorded as 0 if no cells were stained, 1 for <10% of stained cells, 2 for 11–50% of stained cells, 3 for 51–80% of stained cells, and 4 for more than 80% of stained cells. The above two scores were multiplied and an IRS ranged from 0 to 12 was generated. All the GC patients were then divided into two groups: PLK1 negative (IRS<6) and PLK1 positive (IRS≥6) [15].

### Statistical analysis for meta-analysis

All microarray or RNA-seq datasets downloaded from public databases were log2 transformed for further analysis. First, we organized the expression level of PLK1 in carcinomas and controls of each record and presented them as the means ± SD (standard deviation). The PLK1 expression pattern of each data set was visualized by scatter plots and ROC diagrams. Then, the pooled SMD with 95% CI was calculated. An observed SMD>0 favored that PLK1 had a higher expression level in cancerous than that in non-cancerous samples, and statistical significance could be considered if the 95% CI did not cross 0. To further determined the ability of PLK1 in differentiating GC tissues from controls, SROC curve was generated and AUC was obtained with the sensitivity and specificity. The value of PLK1 expression on GC clinical outcome was evaluated through the pooled HR and its 95% CI. A pooled HR>1 illustrated a poor prognosis in GC patients with overexpression of PLK1. Meanwhile, 95% CI did not overlap 1, suggesting a significant association. ORs with 95% CI were used to elucidate the relationship between PLK1 expression and clinicopathological parameters.

Chi-squared-based Q-test and I-square (I^2^) tests were carried out to analyze the heterogeneity across studies [48]. I^2^ >50% or a P value less than 0.05 indicated heterogeneity among studies. We selected the method of a fixed effect model or a random effect model according to the heterogeneity analysis. To ensure the robustness of results, sensitivity analyses were performed by sequentially omitting individual studies to evaluate the impact of each dataset on the pooled results. Additionally, Begg's funnel plots and Egger's test were applied to determine the publication bias. If P>0.05, there was no significant publication bias. All above calculations were calculated by SPSS 20.0 (IBM, New York, USA) and Stata12.0 (Stata Corporation, College Station, TX, USA).

### The relevant genes of PLK1 and functional enrichment analysis

The online database GEPIA (http://gepia.cancer-pku.cn/index.html) is an interactive web server for analyzing the RNA sequencing expression based on TCGA and the GTEx projects [49]. We obtained a series of genes with similar expression patterns to PLK1 in gastric cancer via the database.

The gene ontology (GO) analysis was performed for the functional annotation of these related genes. The pathways that the related genes mainly participated in were investigated by KEGG pathway analysis. GO terms and pathways with a P value < 0.05 were significant. Both GO and KEGG pathway analyses were carried out in the Database for Annotation, Visualization and Integrated Discovery (DAVID). Enrichment maps visualizing the results were drawn by R software.

### PPI network analysis and the hub genes

The online STRING 10.5 database (https://string-db.org/) is commonly used to analyze protein interactions. We constructed the PPI network by using the 14 similar genes which were enriched in the pathway of cell cycle. Hub genes were identified according to the numerical digit of the degrees of each node. And we obtained the matrix plots of the expression level of hub genes from TCGA via the online database GEPIA. The scatter plots of correlation between PLK1 and hub genes were also computed by GEPIA.

### Gene set enrichment analysis

To further understand PLK1-related canonical pathways and biological processes in GC, GSEA was carried out (http://www.broad.mit.edu/gsea) [50]. All GC patients in the TCGA cohort were distributed into two groups based on the median expression value of PLK1 and the expression level of PLK1 was used as the phenotype label. For use with GSEA software, the collection of annotated gene sets of h.all.v6.0.symbols.gmt was chosen as the reference gene sets. FDR < 0.01 was used as the cut-off criteria.

## Abbreviations

AUC: area under the curve
BP: biological process
CC: cellular component
CI: confidence interval
CNKI: China National Knowledge Infrastructure
DAVID: Database for Annotation, Visualization and Integrated Discovery
FN: false negative
FP: false positive
GC: gastric cancer
GEO: Gene Expression Omnibus
GEPIA: Gene Expression Profiling Interactive Analysis
GO: gene ontology
GSEA: Gene set enrichment analysis
IHC: immunohistochemistry
KEGG: Kyoto Encyclopedia of Genes
MF: molecular function
OR: odds ration
PLK1: Polo-like kinase 1
PPI: Protein–protein interactions
SMD: standard mean deviation
SROC: summary receiver operating characteristic
TCGA: The Cancer Genome Atlas
TP: true positive
TN: true negative

## References

[1] Ang TL, Fock KM (2014). Clinical epidemiology of gastric cancer. Singapore Med J.

[2] Siegel RL, Miller KD, Jemal A (2017). Cancer statistics, 2017. CA Cancer J Clin.

[3] Kashihara H, Shimada M, Yoshikawa K, Higashijima J, Tokunaga T, Nishi M, Takasu C (2017). Risk factors for recurrence of gastric cancer after curative laparoscopic gastrectomy. J Med Invest.

[4] Lee JH, Kim KM, Cheong JH, Noh SH (2012). Current management and future strategies of gastric cancer. Yonsei Med J.

[5] Chen J, Gong TT, Wu QJ (2016). Parity and gastric cancer risk: a systematic review and dose-response meta-analysis of prospective cohort studies. Sci Rep.

[6] Xu M, Zhou H, Zhang C, He J, Wei H, Zhou M, Lu Y, Sun Y, Ding JW, Zeng J, Peng W, Du F, Gong A (2016). ADAM17 promotes epithelial-mesenchymal transition via TGF-beta/Smad pathway in gastric carcinoma cells. Int J Oncol.

[7] Lei YY, Huang JY, Zhao QR, Jiang N, Xu HM, Wang ZN, Li HQ, Zhang SB, Sun Z (2017). The clinicopathological parameters and prognostic significance of HER2 expression in gastric cancer patients: a meta-analysis of literature. World J Surg Oncol.

[8] Zhang M, Dong Y, Liu H, Wang Y, Zhao S, Xuan Q, Wang Y, Zhang Q (2016). The clinicopathological and prognostic significance of PD-L1 expression in gastric cancer: a meta-analysis of 10 studies with 1,901 patients. Sci Rep.

[9] Wu P, Wu D, Zhao L, Huang L, Shen G, Huang J, Chai Y (2016). Prognostic role of STAT3 in solid tumors: a systematic review and meta-analysis. Oncotarget.

[10] Archambault V, Lepine G, Kachaner D (2015). Understanding the Polo Kinase machine. Oncogene.

[11] Kumar S, Sharma AR, Sharma G, Chakraborty C, Kim J (2016). PLK-1: angel or devil for cell cycle progression. Biochim Biophys Acta.

[12] Zhang Y, Wu Z, Liu D, Wang M, Xiao G, Wang P, Sun X, Ren H, Tang SC, Du N (2017). Augmented expression of polo-like kinase 1 indicates poor clinical outcome for breast patients: a systematic review and meta-analysis. Oncotarget.

[13] Sun W, Su Q, Cao X, Shang B, Chen A, Yin H, Liu B (2014). High expression of polo-like kinase 1 is associated with early development of hepatocellular carcinoma. Int J Genomics.

[14] Han DP, Zhu QL, Cui JT, Wang PX, Qu S, Cao QF, Zong YP, Feng B, Zheng MH, Lu AG (2012). Polo-like kinase 1 is overexpressed in colorectal cancer and participates in the migration and invasion of colorectal cancer cells. Med Sci Monit.

[15] Otsu H, Iimori M, Ando K, Saeki H, Aishima S, Oda Y, Morita M, Matsuo K, Kitao H, Oki E, Maehara Y (2016). Gastric cancer patients with high PLK1 expression and DNA aneuploidy correlate with poor prognosis. Oncology.

[16] Cai XP, Chen LD, Song HB, Zhang CX, Yuan ZW, Xiang ZX (2016). PLK1 promotes epithelial-mesenchymal transition and metastasis of gastric carcinoma cells. Am J Transl Res.

[17] Kanaji S, Saito H, Tsujitani S, Matsumoto S, Tatebe S, Kondo A, Ozaki M, Ito H, Ikeguchi M (2006). Expression of polo-like kinase 1 (PLK1) protein predicts the survival of patients with gastric carcinoma. Oncology.

[18] Weichert W, Ullrich A, Schmidt M, Gekeler V, Noske A, Niesporek S, Buckendahl AC, Dietel M, Denkert C (2006). Expression patterns of polo-like kinase 1 in human gastric cancer. Cancer Sci.

[19] Jang YJ, Kim YS, Kim WH (2006). Oncogenic effect of Polo-like kinase 1 expression in human gastric carcinomas. Int J Oncol.

[20] Lan B, Liu BY, Chen XH, Qu Y, Zhang XQ, Cai Q, Zhu ZG (2007). [Polo like kinase 1 expression and prognostic value in gastric carcinomas]. [Article in Chinese]. Zhonghua Wei Chang Wai Ke Za Zhi.

[21] Yao H, Yang Z, Li Y (2010). [Expression of checkpoint kinase 1 and polo-like kinase 1 and its clinicopathological significance in benign and malignant lesions of the stomach]. [Article in Chinese]. Zhong Nan Da Xue Xue Bao Yi Xue Ban.

[22] Min T, Bing X, Lan L, Haijian W, Min Z, Wei L (2014). Expression and biological characteristics of PLK1 in human gastric carcinoma. Chin J Clin Lab Sci.

[23] Yihua S, Hong Z, Lan L, Min T, Min Z, Wei L, Yuping P (2014). Expression and significance of PLK1 and β-catenin-STAT3 pathway in patients with gastric carcinoma. hin J Clin Lab Sci.

[24] Dan X (2014). Expression of Nek2, Plk1 and Cdk1 in gastric carcinoma and their clinicopathological significance. J Clin Exp Pathol.

[25] Chonggao Z, Heshun X (2009). Relationship between the expression of Plkl gene and pathobiological behaviors of gastric carcinoma. Chin J Ethnomed Ethnopharm.

[26] Zhang Q, Liu NZ, Hong W, Ni Z, Li XM (2005). Expression of Polo-like kinase 1 and its significance in gastric carcinoma. World Chin J Digestol.

[27] Zhuangwei C, Qibao D, Bing L, Hongsheng Z, Shaoqin C, Aiming G (2009). Expression and significance of Plk1, Raf-1 and Ki-67 in gastric carcinoma. J FuJian Med Univ.

[28] Cao GD, Xu XY, Zhang JW, Chen B, Xiong MM (2016). Phosphorylated mammalian target of rapamycin p-mTOR is a favorable prognostic factor than mTOR in gastric cancer. PLoS One.

[29] Li J, Wang Y, Li QG, Xue JJ, Wang Z, Yuan X, Tong JD, Xu LC (2016). Downregulation of FBP1 promotes tumor metastasis and indicates poor prognosis in gastric cancer via regulating epithelial-mesenchymal transition. PLoS One.

[30] Mo J, Luo M, Cui J, Zhou S (2015). Prognostic value of ERCC1 and ERCC2 gene polymorphisms in patients with gastric cancer receiving platinum-based chemotherapy. Int J Clin Exp Pathol.

[31] Rugge M, Capelle LG, Fassan M (2014). Individual risk stratification of gastric cancer: evolving concepts and their impact on clinical practice. Best Pract Res Clin Gastroenterol.

[32] Pu WY, Zhang R, Xiao L, Wu YY, Gong W, Lv XD, Zhong FY, Zhuang ZX, Bai XM, Li K, Xing CG (2016). Prediction of cancer progression in a group of 73 gastric cancer patients by circulating cell-free DNA. BMC Cancer.

[33] Lee MH, Cho Y, Kim DH, Woo HJ, Yang JY, Kwon HJ, Yeon MJ, Park M, Kim SH, Moon C, Tharmalingam N, Kim TU, Kim JB (2016). Menadione induces G2/M arrest in gastric cancer cells by down-regulation of CDC25C and proteasome mediated degradation of CDK1 and cyclin B1. Am J Transl Res.

[34] Liu Z, Sun Q, Wang X (2017). PLK1, A potential target for cancer therapy. Transl Oncol.

[35] Varki A, Varki NM, Borsig L (2009). Molecular basis of metastasis. N Engl J Med.

[36] Cholewa BD, Liu X, Ahmad N (2013). The role of polo-like kinase 1 in carcinogenesis: cause or consequence?. Cancer Res.

[37] Sparta AM, Bressanin D, Chiarini F, Lonetti A, Cappellini A, Evangelisti C, Evangelisti C, Melchionda F, Pession A, Bertaina A, Locatelli F, McCubrey JA, Martelli AM (2014). Therapeutic targeting of Polo-like kinase-1 and Aurora kinases in T-cell acute lymphoblastic leukemia. Cell Cycle.

[38] Chen XH, Lan B, Qu Y, Zhang XQ, Cai Q, Liu BY, Zhu ZG (2006). Inhibitory effect of Polo-like kinase 1 depletion on mitosis and apoptosis of gastric cancer cells. World J Gastroenterol.

[39] Ruf S, Heberle AM, Langelaar-Makkinje M, Gelino S, Wilkinson D, Gerbeth C, Schwarz JJ, Holzwarth B, Warscheid B, Meisinger C, van Vugt MA, Baumeister R, Hansen M (2017). PLK1 (polo like kinase 1) inhibits MTOR complex 1 and promotes autophagy. Autophagy.

[40] Hao NB, Tang B, Wang GZ, Xie R, Hu CJ, Wang SM, Wu YY, Liu E, Xie X, Yang SM (2015). Hepatocyte growth factor (HGF) upregulates heparanase expression via the PI3K/Akt/NF-kappaB signaling pathway for gastric cancer metastasis. Cancer Lett.

[41] Wu X, Chen Y, Li G, Xia L, Gu R, Wen X, Ming X, Chen H (2014). Her3 is associated with poor survival of gastric adenocarcinoma: Her3 promotes proliferation, survival and migration of human gastric cancer mediated by PI3K/AKT signaling pathway. Med Oncol.

[42] Zang M, Zhang B, Zhang Y, Li J, Su L, Zhu Z, Gu Q, Liu B, Yan M (2014). CEACAM6 promotes gastric cancer invasion and metastasis by inducing epithelial-mesenchymal transition via PI3K/AKT signaling pathway. PLoS One.

[43] Lund-Andersen C, Patzke S, Nahse-Kumpf V, Syljuasen RG (2014). PLK1-inhibition can cause radiosensitization or radioresistance dependent on the treatment schedule. Radiother Oncol.

[44] Yim H (2013). Current clinical trials with polo-like kinase 1 inhibitors in solid tumors. Anticancer Drugs.

[45] O'Neil BH, Scott AJ, Ma WW, Cohen SJ, Aisner DL, Menter AR, Tejani MA, Cho JK, Granfortuna J, Coveler AL, Olowokure OO, Baranda JC, Cusnir M (2016). A phase II/III randomized study to compare the efficacy and safety of rigosertib plus gemcitabine versus gemcitabine alone in patients with previously untreated metastatic pancreatic cancer. Ann Oncol.

[46] Gasparrini A, Armstrong B (2011). Multivariate meta-analysis: a method to summarize non-linear associations. Stat Med.

[47] Tierney JF, Stewart LA, Ghersi D, Burdett S, Sydes MR (2007). Practical methods for incorporating summary time-to-event data into meta-analysis. Trials.

[48] Higgins J, Thompson S, Deeks J, Altman D (2002). Statistical heterogeneity in systematic reviews of clinical trials: a critical appraisal of guidelines and practice. J Health Serv Res Policy.

[49] Tang Z, Li C, Kang B, Gao G, Li C, Zhang Z (2017). GEPIA: a web server for cancer and normal gene expression profiling and interactive analyses. Nucleic Acids Res.

[50] Subramanian A, Tamayo P, Mootha VK, Mukherjee S, Ebert BL, Gillette MA, Paulovich A, Pomeroy SL, Golub TR, Lander ES, Mesirov JP (2005). Gene set enrichment analysis: a knowledge-based approach for interpreting genome-wide expression profiles. Proc Natl Acad Sci U S A.

